# Successful improvement of antibiotic prescribing at Primary Care in Andalusia following the implementation of an antimicrobial guide through multifaceted interventions: An interrupted time-series analysis

**DOI:** 10.1371/journal.pone.0233062

**Published:** 2020-05-15

**Authors:** Rocío Fernández-Urrusuno, Carmen Marina Meseguer Barros, Regina Sandra Benavente Cantalejo, Elena Hevia, Carmen Serrano Martino, Aranzazu Irastorza Aldasoro, Juan Limón Mora, Antonio López Navas, Beatriz Pascual de la Pisa

**Affiliations:** 1 Clinical Unit Primary Care Pharmacy Sevilla, Aljarafe-Sevilla Norte Primary Health Care Area, Andalusian Public Health Care Service, Seville, Spain; 2 Service of Pharmacy, Ouest Primary Health Care Area, Madrid Public Health Service, Madrid, Spain; 3 Promotion and Rational Use of Drugs Service, General Direction of Pharmacy, Andalusian Public Health Care Service, Seville, Spain; 4 Microbiology Service, Hospital San Juan de Dios del Aljarafe, Bormujos, Sevilla, Spain; 5 General Direction of Health Care and Health Outcomes, Andalusian Public Health Care Service, Seville, Spain; 6 Coordination Unit of the Spanish National Action Plan on Antimicrobial Resistance, Spanish Medicines Agency and Health Products, Madrid, Spain; 7 Clinical Unit Camas, Andalusian Public Health Care Service, Camas, Seville, Spain; University of Lincoln, UNITED KINGDOM

## Abstract

**Background:**

Most effective strategies designed to improve antimicrobial prescribing have multiple approaches. We assessed the impact of the implementation of a rigorous antimicrobial guide and subsequent multifaceted interventions aimed at improving antimicrobial use in Primary Care.

**Methods:**

A quasi-experimental study was designed. Interventions aimed at achieving a good implementation of the guide consisted of the development of electronic decision support tools, local training meetings, regional workshops, conferences, targets for rates of antibiotic prescribing linked to financial incentives, feedback on antibiotic prescribing, and the implementation of a structured educational antimicrobial stewardship program. Interventions started in 2011, and continued until 2018. Outcomes: rates of antibiotics use, calculated into defined daily doses per 1,000 inhabitants-day (DID). An interrupted time-series analysis was conducted. The study ran from January 2004 until December 2018.

**Results:**

Overall annual antibiotic prescribing rates showed increasing trends in the pre-intervention period. Interventions were followed by significant changes on trends with a decline over time in antibiotic prescribing. Overall antibiotic rates dropped by 28% in the Aljarafe Area and 22% in Andalusia between 2011 and 2018, at rates of -0.90 DID per year (95%CI:-1.05 to -0.75) in Aljarafe, and -0.78 DID (95%CI:-0.95 to -0.60) in Andalusia. Reductions occurred at the expense of the strong decline of penicillins use (33% in Aljarafe, 25% in Andalusia), and more precisely, amoxicillin clavulanate, whose prescription plummeted by around 50%. Quinolones rates decreased before interventions, and continued to decline following interventions with more pronounced downward trends. Decreasing cephalosporins trends continued to decline, at a lesser extent, following interventions in Andalusia. Trends of macrolides rates went from a downward trend to an upward trend from 2011 to 2018.

**Conclusions:**

Multifaceted interventions following the delivering of a rigorous antimicrobial guide, maintained in long-term, with strong institutional support, could led to sustained reductions in antibiotic prescribing in Primary Care.

## Introduction

Strategies to limit inappropriate antibiotic use and spread of drug-resistant microorganisms have become an increasing priority for health policies and services. Overuse of antibiotics for unnecessary conditions not only promotes antibiotic resistance and loss of protective flora, but also increases the likelihood of preventable drug-related adverse events as well as high costs for Health Care Services [1,2]. Although multidrug-resistant strains of pathogens are increasing in hospital settings, an overall reduction of antimicrobial resistance can only be obtained by addressing the outpatient use of antibiotics [3].

Existing evidence suggests that among strategies aimed to decrease prescription rates of antimicrobials in Primary Care those based on multiple approaches were the most effective [4–13]. When several interventions are overlapping, greater impact is expected [1,13–15]. Antimicrobial stewardship programs (ASP), defined as structured programs to promote the rational use of antibiotics, are becoming increasingly common in Primary Care because their potential benefits in process and patients outcomes [16,17]. A priori, it is not known which specific combined interventions are needed to ensure a sustained reduction on antimicrobial prescriptions. However, one of the common and essential elements for ASP is the availability of high quality guides on antimicrobial therapy [4,16,18,19]. High quality antimicrobial guides help clinicians by decreasing uncertainty, improving knowledge, making better diagnoses, and also, they can be used to make shared decisions with patients. An ASP must work to achieve a good implementation of the antimicrobial guides in clinical practice, which will turn into a better compliance with recommendations for treating most common infections: increasing guide-concordance is what leads to a reduction, and better use of antimicrobials [4,16,18].

In the absence of regional or national reference guidelines addressing the most common infections in the community on the websites of Clinical Practice Guidelines of the Andalusian Health Service (AHS) [20] or of the Spanish National Health System [21], we published “The Aljarafe Antimicrobial Therapeutic Guide” [22] in 2011. The publication of the guide was the starting point for carrying out a set of interventions aimed to promote the prudent use of antibiotics. That same year, the European Commission urged the European Parliament and all member states about the urgent need to implement action plans against the rising threats from antimicrobial resistance [23].

The guide met several requirements to be used as a resource in programs for optimizing the use of antimicrobials: rigorous on development and editorial independence (met AGREE criteria [24]), reliability, applicability, accessibility, and continuous updating. Due to these characteristics, the guide was adopted in 2012 by the AHS as a reference for the antimicrobial use improvement strategies and, in 2017, by the Spanish Action Plan on Antimicrobial Resistance, as the national antimicrobial reference guide [25].

The aim of this study was to assess the impact of “The Aljarafe Antimicrobial Therapeutic Guide” publication, and subsequent interventions aimed at improving antibiotics prescribing in the Andalusian Primary Health Care setting. Interrupted time-series analysis (ITSA) was used to determine whether there was a significant change in antibiotic prescribing rates and trends after interventions.

## Methods

We conducted a quasi-experimental study in the Primary Health Care setting, with ITSA. Initially, interventions aimed at implementing the guide were planned to be carried out at the local level at the Aljarafe Primary Health Care Area (a rural and suburban area with a total population of 386 444 inhabitants assisted in 39 Centres). However, shortly thereafter, the AHS adopted the guide as a reference for the implementation of programs to improve the use of antimicrobials in the whole Andalusian Public Health Care System (which serves a population of 8 384 408 inhabitants, assisted in 1,686 Centres). Then, AHS carried out activities to implement the guide throughout the whole region. Further, the guide was incorporated into the website of the Clinical Practice Guidelines in the Spanish National Health System, Guiasalud [21] in 2011, making it accessible throughout the national territory and internet.

### Interventions

From 2009 to 2011, a multidisciplinary team composed by individuals from all relevant professional groups (Primary Care physicians, pediatricians, epidemiologists, dentists, doctors of several hospital specialties, pharmacists, microbiologists, geriatricians, documentalists, etc) developed the antimicrobial guide for the treatment of infectious diseases and prophylaxis in the community [22]. The guide included recommendations for special patient groups, such as those assisting in nursing homes, urgent care, and dental practice, considering relevant host factors: age, comorbidities, renal and hepatic function, pregnancy, breastfeeding, allergies, or risk factors for antimicrobial resistance. Guidelines included diagnostic criteria for each condition, non-pharmacological management, resistance data of pathogens in the community, antimicrobials safety alerts, notifiable diseases, information of interest for Public Health, referral criteria to hospital, nursing care, and other tools to optimize the management of infections, such as delayed prescribing or patient education. The guide was developed as an electronic decision support tool, and it was easy to access and navigate, providing a rapid clinical decision support.

Interventions aimed at disseminating the guide started on 2011, just after the publication of the first edition in both, book and electronic format. The second edition, was published online in 2012, and included safety alerts associated with antimicrobials. The third edition, published online, was undertaken to revise all the recommendations. The update resulted in the recommendation of more restrictive use of antibiotics (mainly of broad spectrum antibiotics) and shorter antibiotic therapies than earlier versions, in line with new evidence. The third edition was published by chapters as they were being updated. This process began in 2016, and was completed in 2018.

Interventions carried out following the delivering of the guide at the regional level included: educational meetings, and institutional training workshops on ASP for general practitioners, paediatricians, dentists, and primary care pharmacists, the development and selection of indicators for continuous monitoring of antimicrobials [26], monthly feedback on antibiotic prescribing to clinicians on their practice, incorporation of targets for rates of antibiotic prescribing linked to financial incentives in pay-for-performance programs, and later, the implementation of a structured educational ASP [27]. At the local level, in the Aljarafe Area, other interventions included in the local implementation plan were carried out, in addition to those implemented at the regional level. These interventions were described in more detail in a previous publication [28]. Additional details regarding interventions, specifying the professionals who performed each action and institutional support, can be found in Table 1, and a timeline is provided in the (**S1 Fig**).

**Table 1:** Summary of main interventions to improve the use of antibiotics in Primary Care.

Year	Institutional support	Who designed and/or performed the interventions	Description
**a) Activities at the local level (Aljarafe Area)**			
2011	Spanish Ministry of Health	Guide coordinating group^a^ and local Primary Care and Hospital managers	First publication of the antimicrobial therapeutic guide (book form and online).
	(Instituto de Salud Carlos III); European Development Regional Found (FEDER); Andalusian Regional Ministry of Health, Andalusian Health Technology Assessment Agency (AETSA)		Official presentation of the guide. Interdisciplinary conference, chaired by Primary Care and Hospital managers, with the participation of renowned speakers in the field of infectious diseases. Free delivery of guidelines for all attendees. The guide was sent by mail to all Primary Care Districts and Hospitals of the Andalusian Health Service.
		Local Primary Care and Hospital managers	Incorporation of the electronic version of the guide to the websites of Primary Care Centers and Hospital.
		Guide implementing group^b^	First dissemination local plan of the guide. Educational meetings in Primary Care Centers (for general practitioners and paediatricians), Dental Care Unit, Emergency Unit, nursing homes with more than 80 residents, and Hospital Units (Emergency Service, Medicine Service, Urology Service, Ophtalmology Service) summarizing the importance of antimicrobial drug resistance, advantages of having a reference guide, the process of elaboration of the guide and participants, the description of the guide’s methodology and contents. For more details, look at ref 28. Number of meetings: 23. Duration of each activity: 1 hour.
2012	Spanish Ministry of Health	Guide coordinating group^a^	Publication of second edition of the guide (online).
	(Instituto de Salud Carlos III); European Development Regional Found (FEDER); Andalusian Regional Ministry of Health, Andalusian Health Technology Assessment Agency (AETSA)	Guide implementing group^b^	Second dissemination local plan. Educational meetings in Primary Care Centers and Hospital units (Gynecology, Spinal Injuries Unit, Pediatric Emergency Service, Otorhinolaryngology Service). For more details, look at ref 28. Content: recommendations for the treatment of infectious diseases in the community (mainly respiratory, urinary, skin, and dental infections) in the form of clinical cases. Number of meetings: 17. Duration of each activity: 1 hour.
		Guide coordinating group^a^; and local Primary Care and Hospital managers	Incorporation of the guide to the Digital Medical History as an electronic decision support tool.
2013	Spanish Ministry of Health	Guide implementing group^b^ (microbiologists)	Third dissemination local plan. Educational meetings in Primary Care Centers. Content: contribution of the Microbiology Laboratory in the implementation of ASP in Primary Care, correct sampling for microbiological diagnosis, interpretation of antibiograms, local resistance data. Number of meetings: 9. Duration of each activity: 1 hour.
	(Instituto de Salud Carlos III); European Development Regional Fund (FEDER); Andalusian Regional Ministry of Health.		
2016–2018	Andalusian Regional Ministry of Health; Andalusian Health Technology Assessment Agency (AETSA)	Guide coordinating group^a^	Publication of third edition of the guide (online).
2018	Andalusian Regional Ministry of Health; Andalusian Health Technology Assessment Agency (AETSA); Spanish Medicines Agency and Health Products (AEMPS)	Guide coordinating group^a^ Local Primary Care and Hospital managers.	Official presentation conference, chaired by local Primary Care managers, Hospital managers, and regional and national authorities summarizing the role of the Aljarafe Guide as reference of regional ASP, and the Spanish Action Plan on Antimicrobial Resistance.
		PIRASOA program Coordinator.	
		Spanish Action Plan on Antimicrobial Resistance Coordinator	
**b) Activities at the regional level (Andalucia)**			
2011	Andalusian Regional Ministry of Health; Andalusian Health Service (AHS).	General Direction of Health Care-AHS, and General Direction of Pharmacy-AHS	Official presentation of the guide: webcast through the portals of the AHS, Andalusian Center for Drug Information (CADIME), Andalusian Patient Safety Observatory, Andalusian website for Rationale Use of Drugs (Taqwin), website of the Agency for Health Technology Assessment (AETSA).
			Incorporation of the guide to website of Clinical Practice Guidelines of the Andalusian Health Service.
2012	Andalusian Regional Ministry of Health: Andalusian School of Public Health (EASP), and Andalusian Patient Safety Observatory; Andalusian Health Service (AHS).	Teaching team^c^	Workshop for Primary Care and Hospital doctors, Primary Care and Hospital pharmacists, and microbiologists, members of the infection teams and commissions for the proper use of antibiotics. One of the objectives or this workshop was to form a group of experts at the regional level, to design activities related to the proper use of antimicrobials. Content: the management of most common infections and antibiotic prescribing, and key aspects about the implementation of Antimicrobial Stewardship Programmes (ASP). Duration of workshop: 8 hours. Workshop were centralized, performed at the EASP, and designed as “training of trainers”, so that the attendees could reproduce it in their Primary Care Centers and Hospitals. To this aim, attendees received a CD with the contents of the presentations and specific bibliography.
		General Direction of Health Care-AHS and General Direction of Pharmacy-AHS	
		Teaching team^d^	Workshop (3 in total) for general practitioners, Primary Care paediatricians and Primary Care pharmacists. Content: the management of most common infections in the community and antibiotic prescribing, taking as reference “The Aljarafe Guide”, and practical aspects about the implementation of ASP in Primary Care. Duration of workshop: 8 hours. Workshops were centralized, performed simultaneously in Seville, Granada and Antequera, and were designed as “training of trainers”, so that the attendees could reproduce it in their Primary Care Centers. To this aim, attendees received an email with the contents of the presentations and specific bibliography.
		Guide coordinating group^a^	
		General Direction of Health Care-AHS and General Direction of Pharmacy-AHS	
			Workshops (3 in total) for Primary Care Pharmacists. Content: teachers provided knowledge, skills and experiences about the issues of antimicrobial resistance, and monitoring and evaluation of antimicrobial prescribing. Duration of workshops: 8 hours. Workshops were centralized, performed at the EASP, and designed as “training of trainers”, so that the attendees could reproduce them in their Primary Care Centers. To this aim, attendees received an email with the contents of the presentations and specific bibliography.
			Design and selection of indicators for monitoring the impact of ASP in Primary Care [26]
		Local Primary Care and Hospital managers	Incorporation of the guide (or an adaptation) to the Digital Medical History as an electronic decision support tool.
2013	Andalusian Regional Ministry of Health; Andalusian Health Service (AHS)	General Direction of Pharmacy-AHS	Incorporation of targets for antibiotic prescribing indicators linked to financial incentives in the annual agreements signed by Primary Care Areas and Clinical Units with the AHS.
		General Direction of Pharmacy-AHS	Monthly monitoring and feedback to managers and prescribers in Primary Care (general practitioners, paediatricians and dentists) on aggregated and individual rates of antibiotic prescribing. Benchmarking.
		Primary Care Pharmacy Services	
2014	Andalusian Regional Ministry of Health	PIRASOA program Committee.	Implementation of institutional Antimicrobial Stewardship Program (ASP) (PIRASOA). Educational ASP comprised the creation of local antimicrobial multidisciplinary teams responsible for implementing interventions. The core of the programme was the counselling interviews. PIRASOA programme took “The Aljarafe Antimicrobial Therapeutic Guide” as reference guide for Primary Care. Since them, dissemination of Aljarafe guide updates was provided through the PIRASOA program. The PIRASOA elements can be consulted elsewhere [27, 29].
		Local ASP PIRASOA teams	
**c) Other activities at the regional or national level that may influence the rate of antimicrobials prescribing**			
**Year**	**Who performed the intervention**		**Interventions**
2011	Spanish Ministry of Health		Incorporation of the guide to the website of the Spanish National Health System (Guiasalud) [21]
2013	Spanish Ministry of Health; Spanish Agency for Medicines and Medical Devices (AEMPS)		Change of antimicrobials packaging [30]
2014	Spanish Ministry of Health; Spanish Agency for Medicines and Medical Devices (AEMPS)		Spanish Action Plan on Antimicrobial Resistance starts [25].
2016	Andalusian Ministry of Health		Introduction of 13-valent pneumococcal conjugate vaccine, in the Andalusian programme of childhood vaccination.
2017	Spanish Agency for Medicines and Medical Devices (AEMPS)		Spanish Action Plan on Antimicrobial Resistance adopted Aljarafe Guide as reference for ASP in Primary Care.

^a^Guide coordinating group: a Primary Care Pharmacist, a Microbiologist, a Hospital Pharmacist, all of them, experts in infectious diseases and antibiotic management.

^b^Guide implementing group: three Microbiologists, two Primary Care Pharmacists, two Hospital Pharmacists, four specialists in Family and Community Medicine (one of them from the Emergencies Hospital Service), two Pediatric specialists (one from Primary Care and the other from Pediatric Hospital Emergencies), two specialists in Internal Medicine, a specialist in Otorhinolaryngology, a dentist. They were all part of the team that developed the guide.

^c^Teaching team: two members of the General Direction of Health Care (Andalucian Health Service (AHS)), the AHS General Director of Pharmacy, the Head of Service of Rational Drug Use (AHS), a Primary Care Pharmacists, an Infectious Diseases specialist. These professionals were selected by the AHS General Direction of Health Care and General Direction of Pharmacy for their expertise in infectious diseases and antibiotic management.

^d^Teaching team: two members of the General Direction of Health Care (Andalucian Health Service (AHS)), the AHS General Director of Pharmacy, the Head of Service of Rational Drug Use (AHS), three Primary Care Pharmacists, three specialists in Family and Community Medicine; three Microbiologists, a Hospital Pharmacist, an Infectious Diseases specialist. These professionals were selected by the AHS General Direction of Pharmacy for their expertise in infectious diseases and antibiotic management.

The guide was applicable in Primary Care (family practices, paediatric practices, emergency rooms, and dental care units), in hospital emergency rooms, and in nursing homes, to treat ambulatory patients with infectious diseases.

### Data sources and measures

Aggregated antibiotic prescribing data (ATC J01 class) were extracted from the computerised pharmacy records of dispensed drugs, reimbursed by AHS. Authors accessed aggregated data, meaning that individual patients could not be identified. Antimicrobial consumption rates were expressed into defined daily doses (DDD) per 1,000 inhabitants-days (DID) by using the DDD in force until the end of the study. Population data were obtained from the National Statistics Institute database [31].

Consumption of the following classes of antibiotics was measured: overall rate of antibiotic prescribing (DID J01, antibacterials for systemic use) (this indicator was included in pay-for performance programs by the AHS from 2013); DID beta-lactam antibacterials, penicillins (J01C); DID other beta-lactam antibacterials, cephalosporins (J01D); DID quinolones (J01M); DID macrolides, lincosamides and streptogramins (J01F) (when we refer to this indicator, we will refer to as “macrolides”, since it represents almost the total prescription in this group), and DID amoxicillin and betalactamase inhibitor, amoxicillin clavulanate (J01CR02). When we refer to broad-spectrum antibiotics, we refer to amoxicillin clavulanate, quinolones and 3rd generation cephalosporins. Penicillins, amoxicillin, macrolides, or nitrofurantoin are considered narrow-spectrum antibiotics. Data for each year were collected during the following year.

### Analysis

The study ran from January 2004 until December 2018. A sensitivity analysis was done to choose the best cut point of trends in terms of antibiotic prescribing rates. ITSA was carried out using various time points, including 2010, 2011, 2012, and 2013, for the total consumption of antibiotics in both series, Aljarafe and Andalusia. This led as to identify 2011 as the turning point, i.e. the time when a change in trends in total antibiotic prescribing rates was observed. Prescribing data were collected over 7 years pre-interventions (baseline) and 8 years post/during interventions. We conducted ITSA to determinate whether there was a measurable change in level and/or trends of antibiotic prescribing rates after interventions. ITSA was conducted with “ITSA module” of Stata Programme, which produces Newey-West standard errors for coefficients estimated by ordinary least squares regression [32]. Gradients of the resulting regression lines were reported, as annual changes in outcomes. Autocorrelation was assessed by computing the Durbin-Watson statistic. Since evidence of autocorrelation was detected, all analyses were performed with correction for first or second order autocorrelation (according to the series). To ensure that we fit a model that accounts for the correct correlation structure, we used *actest*. A p-value less than 0.05 was considered significant.

Alternatively, we estimated the effect of interventions by calculating absolute and relative differences between predicted values, expected if interventions had not happened, and observed values. These differences were calculated in 2018, seven years after starting the interventions (2018).

Statistical analysis was performed using Stata v.14 (StataCorp 2015. Stata Statistical Software: Release 14. College Station, TX: StataCorpLP).

### Ethics statement

This research was conducted in accordance with the Declaration of Helsinki and national and institutional legislation in Spain regarding clinical research and personal data protection. It has been approved by the Ethics Committee on Health Research of the Hospital Virgen del Rocío (Seville, Spain) (Codes 2010PI/14 and 2012PI/171).

## Results

### Change in annual antibiotic prescriptions in Primary Care

The absolute number of annual DDD antibiotic prescriptions ranged, during pre-intervention period from 2,042,280 in 2004 to 2,750,132 in 2011 in the Aljarafe Area, and from 54,575,612 in 2004 to 64,475,525 in 2011 in Andalusia. Seven years after starting the interventions, DDD prescriptions decreased to 2,078,057 in the Aljarafe Area and to 50,190,292 in Andalusia. Population size increased in this period of time both in Aljarafe (from 314,342 inhabitants in the Aljarafe Area in 2011 to 386,444 in 2018) and Andalusia (from 7,687,518 inhabitants in 2011 to 8,384,408 in 2018).

The overall annual antibiotic DID in Primary Care ranged from 17.89 (in 2004) to a maximum of 20.43 (in 2011) in the Aljarafe Area. In Andalusia, annual antibiotic DID ranged from 19.65 (in 2004) to a maximum of 20.97 (in 2011). Overall, the annual antibiotic prescribing rate dropped by 28% in the Aljarafe Area and 22% in Andalusia, between 2011 and 2018.

The most frequently prescribed antibiotics in 2004 were penicillins, and specifically amoxicillin clavulanate, followed by quinolones, cephalosporins, and macrolides.

Penicillins prescribing rates increased in the pre-intervention period, both in Aljarafe and in Andalusia. Penicillins DID ranged from 12.57 in 2004 to 16.08 in 2011 in Aljarafe, and from 12.38 in 2004 to 14.89 in 2011 in Andalusia. Total penicillins prescribing dropped by 33% in Aljarafe and 25% in Andalusia between 2011 and 2018. Rates of amoxicillin clavulanate changed as follows: they ranged from 7.29 DID in 2004 to 9.76 in 2011 in Aljarafe, and from 8.44 in 2004 to 10.19 in 2011 in Andalusia. Amoxicillin clavulanate DID dropped by 58% in Aljarafe, and 49% in Andalusia between 2011 and 2018.

Quinolones, macrolides, and cephalosporins prescribing rates decreased in the pre-intervention period, both in Aljarafe and Andalusia. Quinolones DID dropped by 51% in Aljarafe and 30% in Andalusia between 2011 and 2018. Cephalosporins DID dropped by 16% in Andalusia between 2011 and 2018. No changes in Aljarafe were observed between these two years. Macrolides DID increased 14% in Aljarafe and 7% in Andalusia between 2011 and 2018.

### Interrupted time series analysis

**Fig 1:**
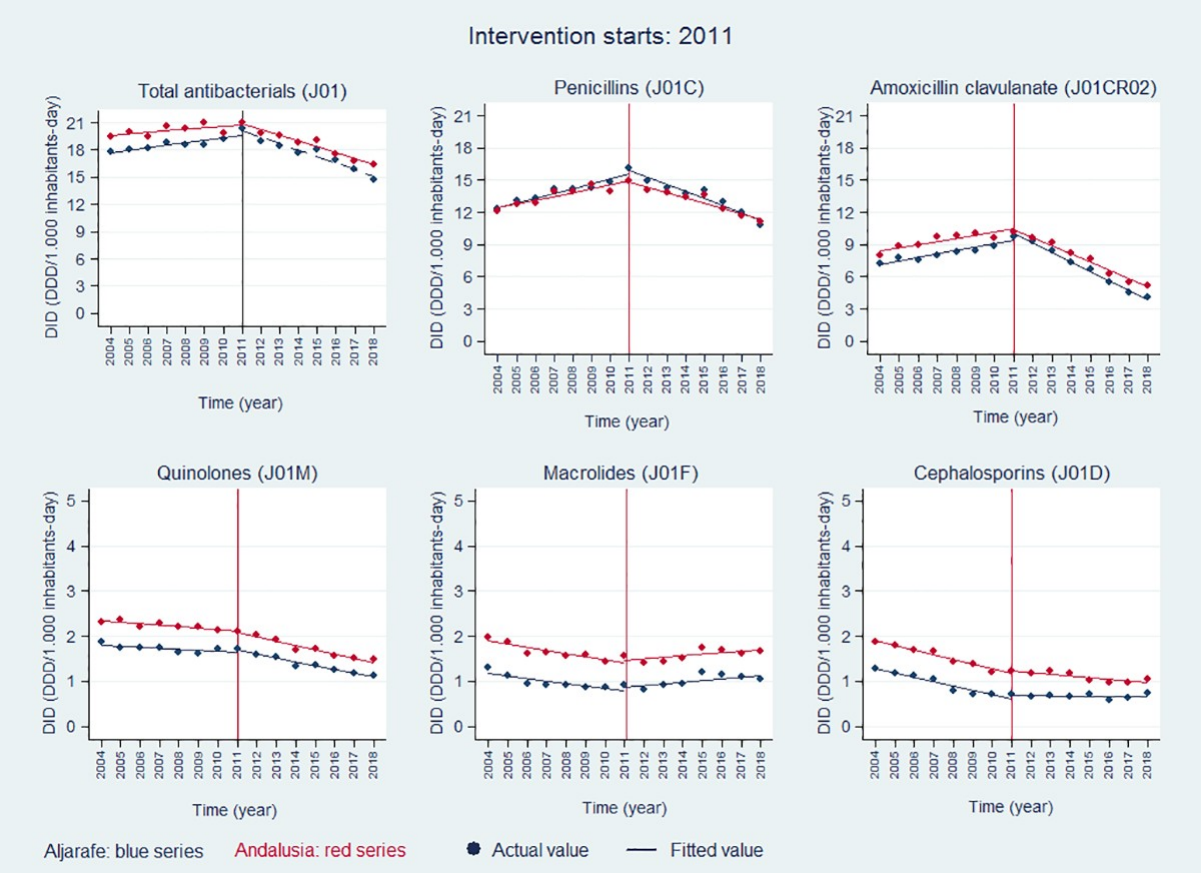
Evolution of annual antibiotic prescribing rates.

ITSA is shown in **Table 2 and Fig 1.** Prior to interventions, trends of overall antibiotic prescribing rates showed a significant increase of 0.19 DID per year in the Aljarafe Area, and a non-significant increase of 0.15 DID per year in Andalusia between 2004 and 2011 (**Table 2**). Trends of total penicillins and amoxicillin clavulanate prescribing increased in the pre-intervention period, both in Aljarafe and Andalusia. Trends in rates of quinolones, cephalosporins, and macrolides decreased in this period, both in Aljarafe and Andalusia (**Table 2, Fig 1**).

**Table 2:** Interrupted time series analysis of antibiotic use from 2004 to 2018 in the Aljarafe Area and in Andalusia.

	Regression intercept (initial level) DID^a^	Pre-intervention trend (annual change from 2004 to 2010) [95%CI] *p-value*	Change in level (change in first year after start of interventions) [95%CI] *p-value*	Post-intervention trend (change in trend from 2011–2018 relative to 2004–2010) [95%CI] *p-value*	Post-intervention linear trend (annual change from 2011 to 2018) [95%CI] *p-value*	Absolute effect (difference between actual and predicted rates) [95%CI]	Relative effect (%) (relative percent change from the pre-intervention trend) [95%CI]
**DID Total antibacterials (J01)**							
Aljarafe	17.88	0.19 [0.13–0.25] 0.000	0.92 [0.32–1.52] 0.006	-0.90 [-1.05-(-0.75)] 0.000	-0.71 [-0.84-(-0.58)] 0.000	-5.38 [-7.03-(-3.73)]	-25.95% [-35.72-(-17.12)]
Andalusia	19.65	0.15 [-0.00–0.30] 0.053	0.16 [-0.65–0.98] 0.669	-0.78 [-0.95-(-0.60)] 0.000	-0.63 [-0.70-(-0.56)] 0.000	-5.30 [-7.23-(3.22)]	-24.20% [-37.54-(13.13)]
**DID Penicillins (J01C)**							
Aljarafe	12.57	0.39 [0.31–0.47] 0.000	0.60 [-0.03–1.22] 0.060	-1.05 [-1.19-(-0.90)] 0.000	-0.65 [-0.78-(-0.52)] 0.000	-4.79 [-0.24–29.76]	-26.00% [-1.42–49.02]
Andalusia	12.38	0.36 [0.19–0.54] 0.001	-0.03–1.00–0.93] 0.940	-0.86 [-1.06-(-0.67)] 0.000	-0.50 [-0.58-(-0.42)] 0.000	-6.05 [-8.42–3.76]	-34.03% [-56.89-(-17.98)]
**DID Amoxicillin clavulanate (J01CR02)**							
Aljarafe	7.29	0.25 [0.21–0.29] 0.000	0.96 [0.61–1.32] 0.000	-1.11 [-1.19-(-1.05)] 0.000	-0.87 [-0.93-(-0.80)] 0.000	-6.81 [-7.65–6.04]	-61.68% [-75.67-(-51.10)]
Andalusia	8.44	0.29 [0.12–0.46] 0.003	-0.06 [-0.83–0.72] 0.876	-1.06 [-1.27-(-0.85)] 0.000	-0.77 [-0.85-(-0.69)] 0.000	-7.48 [-9.72–5.23]	-58.48% [-100.10-(-32.96)]
**DID Quinolones (J01M)**							
Aljarafe	1.81	-0.03 [-0.06–0.00] 0.045	0.06 [-0.06–0.19] 0.291	-0.05 [-0.09-(-0.02)] 0.003	-0.08 [-0.09-(-0.07)] .000	-0.29 [-0.62–0.04]	-21.32% [-74.70-(2.29)]
Andalusia	2.34	-0.03 [-0.04-(-0.02)] 0.000	-0.03 [-0.11–0.05] 0.419	-0.06 [-0.08-(-0.05)] 0.000	-0.10 [-0.11-(-0.08)] 0.000	-0.45 [-0.67–0.31]	-24.19% [-39.41-(-14.90)]
**DID Macrolides, lincosamides and streptogramins (J01F)**							
Aljarafe	1.20	-0.07 [-0.10–0.03] 0.002	0.16 [-0.00–0.32] 0.051	0.10 [0.06–0.16] 0.001	0.04 [0.01–0.07] 0.015	0.85 [0.42–1.44]	607.14% [-91.09–160.00]
Andalusia	1.92	-0.08 [-0.11-(-0.05)] 0.000	0.12 [-0.05–0.29] 0.144	0.11 [0.08–1.15] 0.000	0.03 [0.01–0.06] 0.015	0.89 [0.49–1.34]	123.61% [-3.26–102.29]
**DID Cephalosporins (J01D)**							
Aljarafe	1.30	-0.11 [-0.13–0.09] 0.000	0.15 [-0.04–0.25] 0.013	0.10 [0.07–0.13] 0.000	-0.01 [-0.02–0.01] 0.527	0.85 [0.53–1.16]	-5.54% [-74.65–11600.00]
Andalusia	1.91	-0.11 [-0.13-(-0.09)] 0.000	0.09 [0.00–0.18] 0.041	0.07 [0.05–1.10] 0.000	-0.04 [-0.05-(-0.02)] 0.000	0.59 [0.35–0.88]	225.77% [-354.00–141.94]

^a^DID: defined daily doses per 1000 inhabitants-day

Following the start of interventions, there were significant changes in trends of antibiotic rates with a decline over time in overall antibiotic DID, penicillins DID, and amoxicillin clavulanate DID, a stronger decline in quinolones DID than that observed during the pre-intervention period, a slower decline in cephalosporins DID than that observed during the pre-intervention period. Finally, there was an increase in trends of macrolides rates, both in Aljarafe and Andalusia between 2011 and 2018 (**Table 2, Fig 1**). Compared with the expected antibiotic prescriptions, based on pre-intervention trends, we observed reductions in DID, at the end of the study period for overall antibiotic prescribing (-5.38 DID in Aljarafe, -5.30 DID in Andalusia), penicillins (-4.79 DID in Aljarafe, -6.05 DID in Andalusia), amoxicillin clavulanate (-6.18 DID in Aljarafe, -7.48 DID in Andalusia), and quinolones (-0.29 DID in Aljarafe, -0.45DID in Andalusia) (**Table 2**).

Rates of cephalosporins and macrolides were higher than that expected if the interventions had not happened (**Table 2**), although cephalosporins rates decreased during the post-intervention period.

## Discussion

This study shows that the publication of “The Aljarafe Antimicrobial Therapeutic Guide” in 2011, and subsequent interventions carried out to improve guide adherence or to improve antibiotic use by other mechanisms, i.e. financial incentives, were followed by a significant reduction in rates of antibiotic prescribing in the Primary Care setting in Andalusia. Overall annual antibiotic prescribing rates, decreased since 2011, and differences between actual and predicted rates persisted until the end of the follow-up period. Prescribing rates of frequently misused broad-spectrum antibiotics such as amoxicillin-clavulanate, quinolones, and cephalosporins, targets for effective ASP, decreased during interventions, although it should be pointed out that quinolones and cephalosporins showed downward trends before starting interventions.

Reductions in antibiotic prescriptions were more pronounced in the Aljarafe Area where the guide was developed, and where additional interventions included in the local implementation plan were carried out. The impact of interventions in the whole region, although with less intensity than in the Aljarafe Area, was similar in terms of the decrease of prescribing rates and decreasing tendencies.

No control group was available, as interventions were carried out throughout the region. However, considering that our population evolved to a more aged population during the study period and that elderly are the age group with a greatest antibiotic consumption, increased antibiotic prescribing rates would be expected. Taking into account that the interventions described in this study have not been carried out in other regions of Spain, we could consider national prescribing data as an external comparator. Available national data showed increasing rates of antibiotic prescribing in the community between 2012 and 2016, and a decrease in 2017 [25,33].

In our setting, reduction of overall antibiotic prescribing rate exceeding 20%, occurred immediately after the publication of the guide at a rate of 0.8–0.9 DID per year, and were significant, both at the local level and in the whole region. Further, lower rates of antibiotics were achieved, while the appropriateness of the antibiotics use, according to the patients and processes treated, improved [28]. The magnitude of the decrease was greater than that observed in other studies conducted in Primary Care (3–15%) [2, 5–10]. These important reductions occurred at the expense of the reduction of the most frequently prescribed group of antibiotics: penicillins, and more precisely, at the expense of the reduction of amoxicillin clavulanate whose prescription has plummeted by around 50%. This means that amoxicillin clavulanate was no longer the most frequently prescribed antibiotic in Primary Care, since 2015 in the Aljarafe area, and since 2017 in Andalusia, being surpassed by other penicillins with a narrower spectrum such as amoxicillin, as recommended by the guide for most common infections [22]. The prescription of other broad-spectrum antibiotics such as quinolones and cephalosporins, continued to decline after interventions. Macrolides were the only analyzed antibiotic group, whose rates increased during the post-intervention period. We do not have an explanation for this, since it is not supported by guide’s recommendations.

It could be expected that the long term impact of these results (lower rates of overall antibiotics, lower rates of broad-spectrum antibiotics, and improved appropriateness) provides better health outcomes, less patient safety issues, and less antimicrobial resistance development.

The guide was the core element of the strategies. The development of high quality, reliable guides with several functionalities (gold standard for quality prescribing, basis for educational activities, electronic decision support tool), a relatively simple method to influence many prescribers, seems to play a crucial role. Health professionals need reliable and independent antimicrobial guides to improve quality prescribing and fight some barriers for making the best decisions for their patients with infections. However, just delivering guidelines is not usually enough to achieve major changes [1,34,35]. Interventions like those carried out in our region, such as the integration of evidence-based antimicrobial guides within the electronic medical records [9,14,15,34–36], active clinical education, the introduction of antibiotic prescribing indicators linked to financial incentives [37], audit and feedback of antimicrobial prescribing [6,9,35], visits by peer academic detailers [8], and the recommendation of delayed prescription strategies in acute uncomplicated respiratory infections [12], have shown good results in increasing guidelines-concordant prescribing and reducing antibiotic prescribing rates in Primary Care.

The size of the effect of the interventions varies considerably and, once several interventions are implemented simultaneously, it is difficult to differentiate the impact of each strategy [1]. We can find very successful experiences (30–43% reductions in overall antibiotic prescribing) [13,38,39], and powerful interventions that have not had the expected results. For example, little success in the reduction of total antibiotic prescribing was observed after the application of an intensive and multifaceted educational program (STAR) in Wales (a 4.2% decrease) [5]. In Switzerland, Hürlimann *et al* did not achieve any reduction in antibiotic prescribing rates after the implementation of antimicrobial guidelines, coupled with feedback on individual antibiotic prescribing [40]. In our region, the implementation of a structured educational institutional ASP in 2014, three years after the start of interventions, have not had an additional impact on trends of overall antimicrobial prescribing rates, rates of broad-spectrum antibiotics prescribing, nor those of narrow-spectrum antibiotics prescribing in Primary Care [29], although it could be helping to keep the trends down.

### Strengths and limitations

This study was large, including a complete geographical region and all prescriptions dispensed from general practitioners, paediatricians and dentists of a Public Health Care Service, thus providing a complete picture of overall antibiotic prescribing in Primary Care. Our findings may be applied to other health services with the same characteristics. On the other hand, we have monitored the impact of interventions of a long enough period of time to establish the sustainability of the effects.

There are several study limitations. Firstly, this is a quasi-experimental study without a control group. We cannot exclude the possibility that factors other than these interventions could influence antibiotic prescribing during the period of the study. To overcome this limitation, we performed an ITSA. ITSA is arguably the strongest quasi-experimental research design and is particularly useful when a randomized trial is infeasible or unethical. Due to the few time points, our results should be interpreted with caution as they may be underpowered. Although we did not have a control group, the availability of national data [25,33] allowed us to have an external control.

Second, the data refer to the prescriptions made in Primary Care of the Public Health Service. There has been no follow-up of the prescriptions to outpatients by Hospital prescribers, where the interventions have not been made.

Third, we assumed a good therapeutic compliance, and that the actual consumption of antibiotics corresponded to the dispensations made.

Finally, we cannot know the actual level of use of the guide in our setting, since one cannot know the number of queries in the book format or the number of downloads in all electronic platforms where it is uploaded. With regard to the electronic version, we cannot differentiate queries that have been made in our setting and those that have been made from other settings.

Being aware of limitations, this study shows that multifaceted and continuous interventions following the delivering of a rigorous, reliable, and accessible antimicrobial guide led to significant and sustained reduction in overall antibiotic prescribing in Primary Care, mainly due to the reduction in penicillins prescribing (especially amoxicillin clavulanate), and other broad-spectrum antibiotics such as quinolones and cephalosporins. This sustained decrease probably resulted from the additive effect of successive interventions maintained over time, with a great institutional support, enabling the incorporation of guide recommendations in everyday clinical practice by a large number of professionals. As the benefit of the interventions reverts after they ceased [14,34,41], when planning strategies aimed at reducing antibiotic prescribing over time, it should be contemplated as part of their long term maintenance. To this aim, the institutional support is crucial, since it is what guarantees a commitment to the implementation in the whole health system, the availability of the necessary resources, and the maintenance of the interventions over time.

These findings have important implications for the Spanish National Health System, given the very high prescription rates of antimicrobials and resistances in our country, compared to those from other European Union countries [33,42]. One of the main objectives of the Spanish Action Plan on Antimicrobial Resistance is the implementation of ASP in the community [25]. Uptake of the Aljarafe guide as reference for ASP has been agreed, since it could result in a substantial reduction of antibiotic pressure throughout the whole national territory.

In conclusion, multifaceted interventions following the delivery of a rigorous antimicrobial guide, maintained in long-term, with strong institutional support, could lead to sustained improvement in antibiotic prescribing in Primary Care. Which specific elements that must be added to multiple strategies at the local setting, will depend on the initial situation, resources, and institutional support without losing sight of the fact that, before starting programs, it is important to know which strategies can be supported by institutions for long-term.

## Supporting information

S1 Fig Timeline of interventions. (PDF)

S1 Data (XLS)
