# Supplementary figures and images for: Successful improvement of antibiotic prescribing at Primary Care in Andalusia following the implementation of an antimicrobial guide through multifaceted interventions: An interrupted time-series analysis
Source: PLoS One. 2020 May 15;15(5):e0233062. doi: 10.1371/journal.pone.0233062 (PMC7228088; doi:10.1371/journal.pone.0233062)

## Publication of the Aljarafe Guide

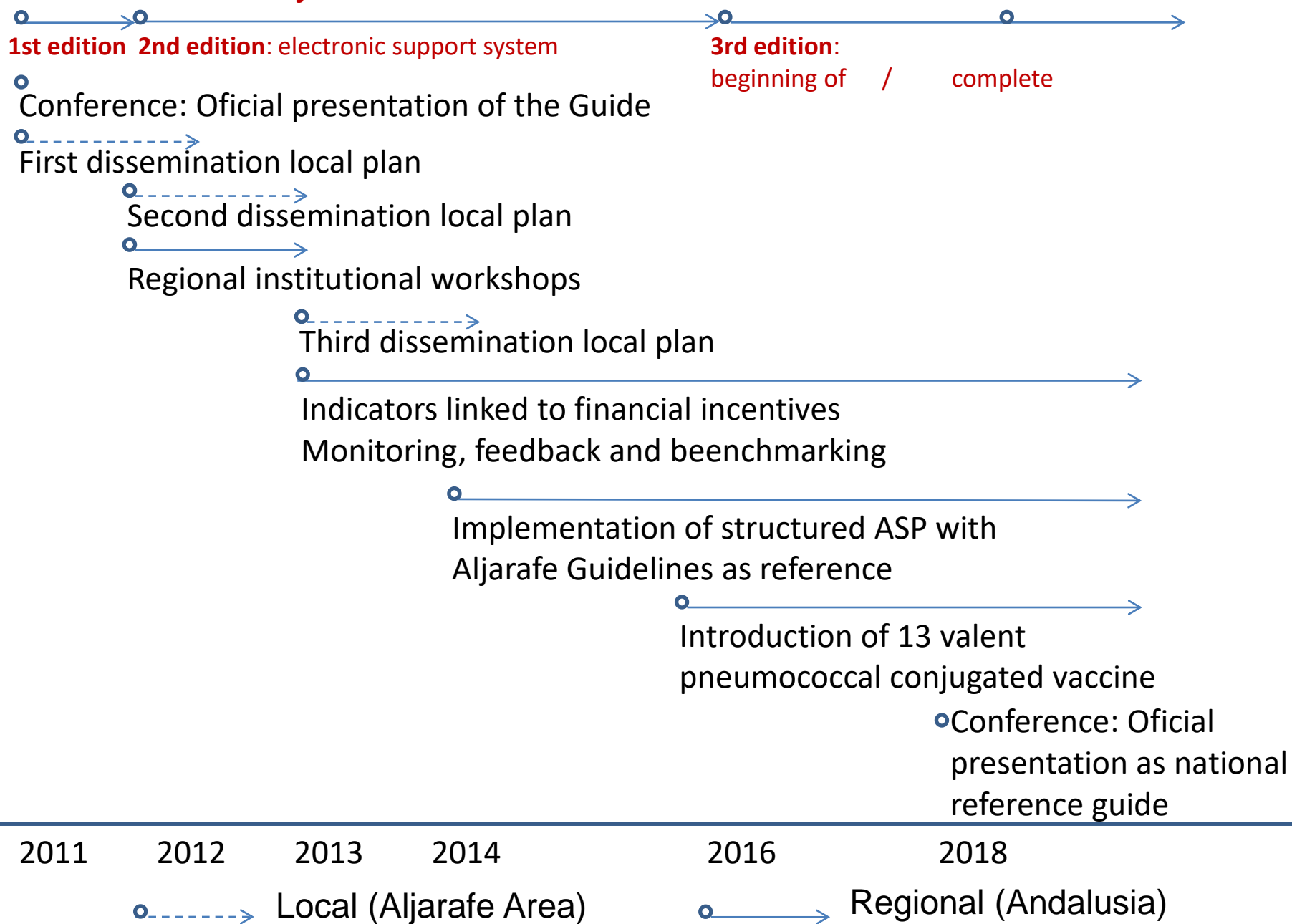

Supplement: S1 Fig — (PDF) [file pone.0233062.s001.pdf]
